# The M2 Macrophages Importance Role in Psoriasis

**DOI:** 10.1002/iid3.70211

**Published:** 2025-06-20

**Authors:** Ahmed Hussein Hasan Alshihmani, Mahmoud Mahmoudi, Ramiar Kamal Kheder, Afsane Fadaee, Seyed‐Alireza Esmaeili

**Affiliations:** ^1^ Immunology Research Center Mashhad University of Medical Sciences Mashhad Iran; ^2^ Immunology Department, Faculty of Medicine Mashhad University of Medical Sciences Mashhad Iran; ^3^ Medical Laboratory Science Department, College of Science University of Raparin Rania Sulaymaniyah Iraq; ^4^ Department of Medical Analysis, Faculty of Applied Science Tishk International University Erbil Iraq

**Keywords:** autoimmune conditions, cytokines, immune regulation, inflammation, M2 macrophages, macrophage polarization, psoriasis, therapeutic interventions

## Abstract

**Background:**

Psoriasis is a chronic autoimmune skin condition that is increasingly prevalent globally, causing significant challenges for affected individuals. The disease is influenced by a combination of genetic factors, environmental triggers, immune system dysfunction, and its systemic nature with comorbidities like cardiovascular diseases and depression. Macrophages, essential immune cells, play a critical role in the pathogenesis of psoriasis, displaying various functions and activation states.

**Objective:**

This study aims to investigate the impact of M2 macrophages on the progression and management of psoriasis, particularly their ability to support tissue healing and reduce inflammation.

**Results:**

M1 macrophages are involved in early inflammation and pro‐inflammatory responses when activated by LPS and IFN‐γ, while M2 macrophages are activated by IL‐4 and IL‐13 to promote anti‐inflammatory and tissue repair processes. Imbalance in M1 and M2 polarization can worsen autoimmune conditions such as psoriasis, underscoring the need to regulate macrophage phenotypes for effective disease management. Recent research suggests that targeting M2 macrophages could be a promising therapeutic approach for treating psoriasis, especially through advanced biologic treatments such as anti‐IL‐17 and anti‐IL‐23 therapies. Enhancing the function of M2 macrophages has been shown to reduce inflammation and improve outcomes in psoriatic conditions, potentially lowering the risk of comorbidities through early intervention and personalized treatment. Biomarkers like CD163(+) M2 macrophages are associated with disease progression, while treatments that enhance M2 macrophage function, such as PSORI‐CM02 and Treg‐of‐B cell therapies, show potential in alleviating psoriasis symptoms.

**Conclusion:**

Understanding the crucial role of M2 macrophages in psoriasis could pave the way for innovative treatment approaches that regulate immune responses and enhance patient care. Further exploration of macrophage biology in psoriasis may provide fresh perspectives on personalized therapeutic options for managing psoriasis and other inflammatory skin conditions, ultimately improving patient care and quality of life for individuals with this chronic skin condition.

## 1 Introduction

Psoriasis is a chronic autoimmune skin condition, affecting approximately 2%–3% of the global population due to genetic predisposition, with significant systemic impacts and comorbidities such as cardiovascular disease and depression [1, 2]. The prevalence of psoriasis has been increasing, making it the second leading cause of skin disabilities worldwide [3]. The disease is characterized by the abnormal growth and differentiation of keratinocytes, the main cells in the outer layer of the skin, resulting in thickened, scaly patches [4, 5]. Psoriasis is characterized by keratinocyte abnormalities, inflammation, and immune cell infiltration, which worsen skin lesions [6, 7]. The primary pathological mechanism of this disease involves the interaction between adaptive and innate immunity, including macrophages, T cells, and dendritic cells interact to regulate inflammation in psoriasis, with macrophage plasticity playing a key role. For example, IFN‐γ drives M1 polarization, amplifying inflammation, while IL‐4 and IL‐13 promote M2 differentiation, which can aid tissue repair but, if dysregulated, may contribute to fibrosis [8, 9]. During this process, different immune cells such as T cells, dendritic cells, keratinocytes, and macrophages communicate by releasing cytokines, which are small proteins that serve as messengers in the immune system [8, 10]. These cytokines help recruit and activate immune cells in the skin, causing ongoing inflammation and persistent, debilitating symptoms in psoriasis patients [10, 11]. Psoriasis is believed to be caused by a combination of genetic and environmental factors [12, 13]. Individuals with a parent who has psoriasis are at a higher risk of developing the condition [14].

## 2 Symptoms and Risk Factors

Symptoms of psoriasis include red patches of skin that develop into erythematous plaques covered with silvery‐white scales. These patches are scaly, itchy, and can be painful [15, 16]. These symptoms can significantly impact a person's quality of life [5, 17]. Psoriasis commonly appears on the elbows, knees, scalp, trunk, buttocks, and extremities, but can occur anywhere on the body [8, 18]. Severe cases of psoriasis can lead to skin lesions, arthritis, and mucosal involvement in areas like the pharynx and gastrointestinal tract, which may cause gastrointestinal bleeding. Additionally, psoriasis is frequently associated with comorbidities such as cardiovascular disease, obesity, and depression [19, 20]. Risk factors for psoriasis are diverse [21]. Genetic predisposition including a family history of the disease, plays a significant role [22, 23]. Specific genetic factors like HLA‐Cw6 and mutations in the CARD14 gene, contribute to the risk [24]. Other risk factors are categorized into extrinsic factors influenced by external conditions such as stress, air pollutants, sun exposure, drugs, vaccination, infection, and lifestyle choices, and intrinsic factors inherent to the individual [10]. These factors collectively trigger or worsen the condition, highlighting the complex interaction between genetics, environment, and lifestyle in managing psoriasis [10, 24]. In genetically predisposed individuals, environmental factors can induce epigenetic changes that impact the disease process [24].

## 3 Importance of Early Diagnosis and Treatment

Early diagnosis and treatment are crucial in effectively managing psoriasis, as recent studies have highlighted. Prompt identification enables healthcare providers to start appropriate therapies quickly, which can greatly reduce symptoms and enhance the patient's quality of life [18, 25, 26, 27]. Delaying the diagnosis and treatment may result in the worsening of symptoms, including severe skin lesions and joint involvement, such as psoriatic arthritis [27]. Early intervention is crucial in managing psoriasis and reducing the risk of developing related conditions such as cardiovascular diseases, diabetes, obesity, inflammatory bowel disease, nonalcoholic fatty liver disease, depression, and anxiety. Recent studies suggest that timely treatment can disrupt the psoriatic march, reducing inflammation and halting disease progression to comorbid conditions like psoriatic arthritis. Research shows that early treatment not only alleviates physical symptoms but also improves mental health, leading to better overall well‐being [18, 27]. Effective treatment strategies for psoriasis include topical therapies, phototherapy, and systemic medications, which can be classified into conventional treatments like methotrexate and cyclosporine, and nonconventional therapies such as biologics targeting TNF‐α and IL‐23. However, long‐term use of immunosuppressants is limited due to side effects like infection. These treatments help reduce inflammation, slow down the overproduction of skin cells, and manage symptoms [26]. In mild‐to‐moderate cases, topical therapies are the primary approach, while they are used adjunctively with systemic therapy in severe cases [28, 29]. Therapeutic advances for moderate to severe plaque psoriasis include biologics targeting TNF‐α, p40IL‐12/23, IL‐17, and p19IL‐23, as well as an oral phosphodiesterase 4 inhibitor [29]. Narrowband UV‐B phototherapy is commonly prescribed for treating plaque psoriasis [29, 30]. Modern dermatology focuses on personalized treatment plans for psoriasis based on severity, type, and individual needs, guided by genetic and molecular biomarkers that help predict treatment response and optimize outcomes. This approach targets inflammation, regulates skin cell turnover, and manages symptoms effectively. Early diagnosis and treatment awareness are essential to minimize long‐term impact on health and quality of life [26, 31, 32].

## 4 Macrophages and Their Classification

Macrophages are derived from monocytes, a type of white blood cell found in the bloodstream [33, 34]. Monocytes are primarily produced in the bone marrow from a common myeloid precursor with neutrophils before entering the bloodstream [35, 36]. Monocytes transition from the bloodstream to tissues in both homeostasis and inflammation [34, 35, 37]. As monocytes migrate into peripheral tissues, they differentiate into mature macrophages in response to local growth factors, pro‐inflammatory cytokines, and microbial products [35, 38]. Macrophages can be classified based on their activation state and function into two main categories: classically activated macrophages (M1) and alternatively activated macrophages (M2) [38, 39]. M1 macrophages are induced by pro‐inflammatory signals such as interferon‐gamma (IFN‐γ) and lipopolysaccharide (LPS), exhibiting pro‐inflammatory and antimicrobial properties [40, 41, 42]. On the other hand, M2 macrophages are induced by anti‐inflammatory signals like interleukin‐4 (IL‐4) and interleukin‐13 (IL‐13) [43, 44]. M2 macrophages play a role in tissue repair, wound healing, and inflammation resolution by producing anti‐inflammatory cytokines such as IL‐10 and TGF‐β and facilitating tissue remodeling. However, dysregulation of M2 macrophages can contribute to pathological fibrosis, which may worsen psoriatic lesions. These macrophages also interact with T cells to regulate immune responses, influencing inflammation and tolerance [40, 45]. Macrophages M2 are classified into four subtypes: M2a, M2b, M2c, and M2d, each serving distinct roles in the immune system in response to different signals. M2a macrophages, activated by IL‐4 and IL‐13, aid in tissue repair and combat parasitic infections [46, 47, 48]. M2b macrophages, triggered by cytokines like IL‐1β and LPS, regulate immune responses [45]. M2c macrophages, induced by IL‐10 and glucocorticoids, suppress inflammation and promote tissue healing [49]. M2d macrophages, influenced by growth factors and specific cytokines, play a crucial role in angiogenesis by promoting the formation of new blood vessels [50]. Imbalances in macrophage M1/M2 polarization can contribute to various diseases or inflammatory conditions. Biomarkers such as CD163, expressed on M2 macrophages, are associated with anti‐inflammatory functions and can serve as indicators of immune regulation in inflammatory diseases like psoriasis. In addition to M1 and M2, macrophages can be further categorized based on their tissue location and specific functions, responding to environmental cues [51, 52] For example, Kupffer cells in the liver aid in blood detoxification, while alveolar macrophages in the lungs clear inhaled particles and pathogens [53, 54]. The diverse functions of macrophage subtypes enable the body to effectively manage different physiological and pathological situations [39, 52]. When mature macrophages are transferred to a new tissue environment, they can adapt their gene expression profiles to perform specific immune functions, showcasing their ability to respond to environmental changes and signals, which can be beneficial in treating various diseases [55, 56].

## 5 The Critical Role of Macrophages in Immune System Mechanisms

Macrophages play a vital role in the immune system by performing key functions in both innate and adaptive immunity. They are responsible for clearing pathogens, dead cells, and debris through phagocytosis, which helps prevent the spread of infections and maintain tissue integrity [39, 44, 49]. Macrophages play a vital role in coordinating the immune response by producing and releasing various cytokines and chemokines that regulate the functions of other immune cells. In inflammatory diseases such as psoriasis, dysregulated macrophage activity can exacerbate immune responses, highlighting their role in autoimmune conditions. For instance, the psoriatic march links systemic inflammation with comorbidities such as psoriatic arthritis, with osteoclast involvement playing a key role [44, 57, 58]. They secrete inflammatory cytokines such as tumor necrosis factor‐alpha (TNF‐α), interleukin‐1 (IL‐1), and interleukin‐6 (IL‐6) to enhance the inflammatory reaction and attract more immune cells to the site of infection [44, 57, 58]. Macrophages act as antigen‐presenting cells (APCs) by presenting antigen fragments on their surface using major histocompatibility complex (MHC) molecules after digesting pathogens. This presentation is essential for activating T cells, which are crucial for adaptive immunity. Activated T cells then multiply and differentiate into various subsets that help eliminate the infection and create long‐lasting immunity [59, 60, 61]. Macrophages are essential for tissue repair and regeneration after immune responses. They release growth factors and anti‐inflammatory cytokines like interleukin‐10 (IL‐10) and transforming growth factor‐beta (TGF‐β) to promote wound healing and reduce inflammation. Biomarkers such as CD163 on M2 macrophages are associated with these anti‐inflammatory functions, serving as potential indicators of disease progression or response to treatment in conditions like psoriasis. However, dysregulated M2 macrophages can contribute to tissue fibrosis, a key factor in chronic inflammation. By clearing apoptotic cells and supporting new tissue growth, macrophages help restore normal tissue function in damaged areas [39, 62, 63]. Macrophages are highly versatile and adaptable cells that respond to different environmental cues and signals. They can switch between fighting infections, presenting antigens, and aiding in tissue repair. This diverse capability highlights the crucial role of macrophages in maintaining the balance and effectiveness of the immune system [39, 44, 49].

## 6 Differences Between M1 and M2 Macrophages and Their Regulatory Factors

**Figure 1:**
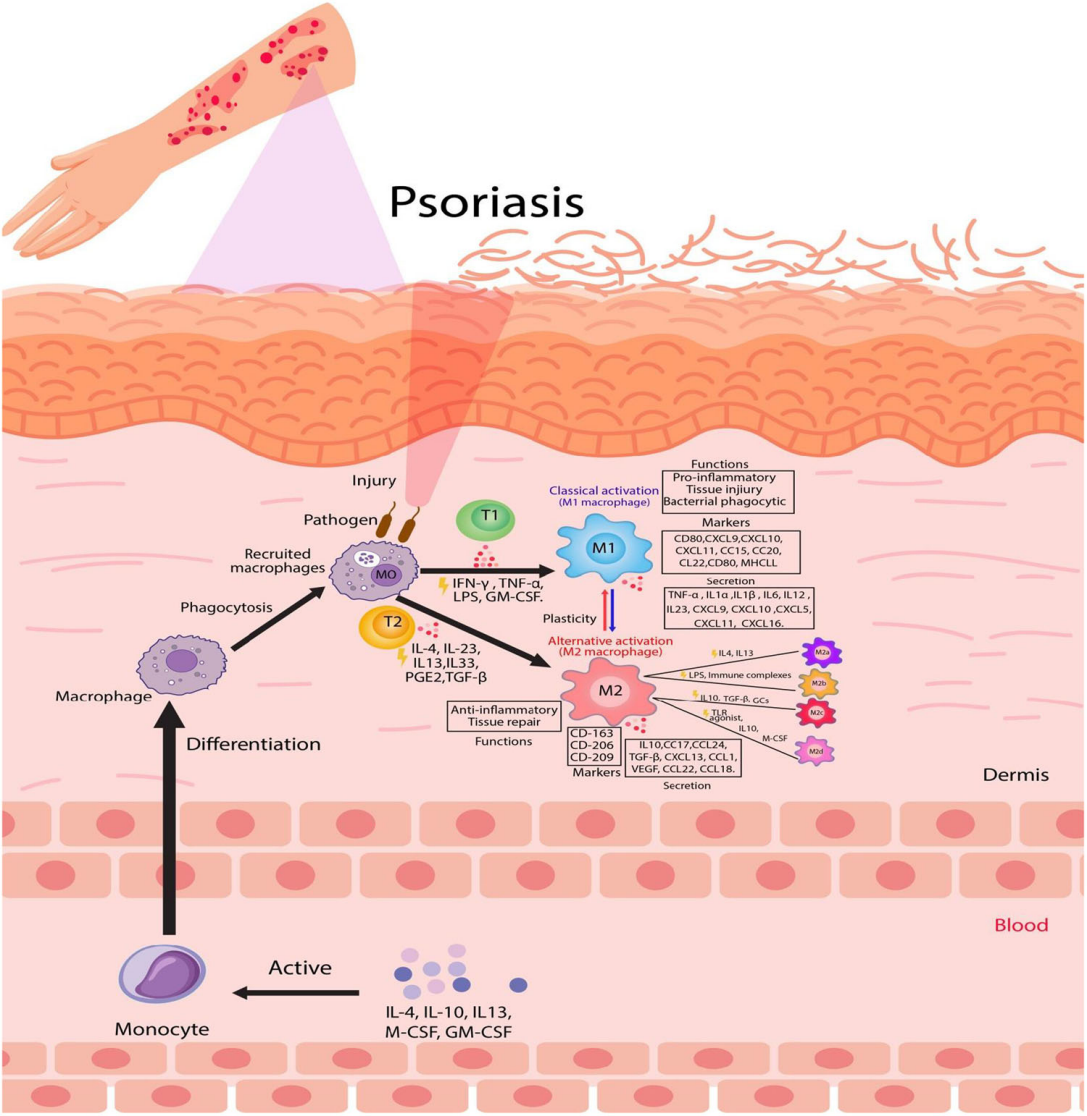
Comparison of M1 and M2 macrophages: regulatory factors and roles in psoriasis.

Table 1 compares M1 and M2 macrophages and their regulatory factors, highlighting their distinct functions and pathways. M1 macrophages are primarily activated by IFN‐γ and LPS, known as classically activated macrophages [41]. CD86 acts as a costimulatory molecule in M1 macrophages, while MHC II facilitates antigen presentation to helper T cells, essential for immune response regulation. Through the activation of various transcription factors [28], M1 macrophages produce inflammatory cytokines like IL‐1β, IL‐6, IL‐12, IL‐23, and TNF‐α, crucial for pathogen and tumor cell elimination [68]. M1 macrophages generate reactive oxygen species (ROS) and nitric oxide (NO), to combat pathogens. These processes are driven by pathways like NF‐κB and STAT1 activation, which promote cytotoxic and pro‐inflammatory functions [77, 78]. They are critical in the early phases of inflammation and aid in the recruitment of additional immune cells to infection or injury sites [79]. These macrophages are physiologically predisposed to glycolysis and play important roles in chronic inflammation and autoimmune conditions [69, 80]. M1 macrophage polarization is driven by NF‐κB and STAT1 activation, leading to cytotoxic and pro‐inflammatory functions [51]. M2 macrophages, also known as alternatively activated macrophages, are stimulated by IL‐4 and IL‐13 [64], express surface markers like CD206 and CD163. These markers, particularly CD163, are critical for monitoring macrophage polarization and inflammation in autoimmune conditions like psoriasis [81, 82], and produce anti‐inflammatory cytokines such as IL‐10, TGF‐β [69, 70]. They promote oxidative phosphorylation and fatty acid oxidation, which aids in tissue healing and tumor growth [44, 83, 84]. M2 macrophages are typically found in healing wounds and adipose tissue, exhibiting stronger phagocytic activity than M1 macrophages [85]. M2 macrophages promote angiogenesis and extracellular matrix deposition, which are critical for wound healing. However, their dysregulation can lead to pathological fibrosis, exacerbating chronic inflammatory conditions like psoriasis [86]. Activation of the STAT3 and STAT6 pathways by IL‐4/13 and IL‐10 polarizes M2 macrophages [51, 87]. PPARδ and PPARγ playing roles in controlling oxidative metabolism. Transcription factor KLF‐4 downstream of STAT6 inhibits NF‐κB/HIF‐1α‐dependent transcription to promote M2 activities [51, 88, 89]. IL‐10 enhances M2 polarization by activating p50 NF‐κB homodimer, c‐Maf, and STAT3 activity [51, 90]. Additionally, microRNAs like miR‐155 and miR‐223 influence macrophage polarization by targeting SOCS1, CEBP, and Pknox1, respectively. The microenvironment and stimuli encountered by macrophages determine their polarization into M1 or M2 phenotypes [48, 51, 91]. A balanced M1/M2 response is crucial for immune control and tissue homeostasis [52, 69], as dysregulation can lead to chronic inflammation and autoimmune disorders, including psoriasis. In psoriasis, an M1‐dominant response amplifies pro‐inflammatory pathways, contributing to disease severity and This highlights the importance of balancing M1/M2 polarization to reduce inflammation and support tissue repair [80, 92]. See Figure 1 for a schematic illustration of the differences between M1 and M2 macrophages, their regulatory factors, and their roles in psoriasis (created using Adobe Illustrator 2020).

**Table 1:** Differences between M1 and M2 macrophages and their regulatory factors.

Feature	M1 Macrophages	M2 Macrophages	Ref
Phenotypes	Classically activated macrophages	Alternativ elyactivated macrophages	[41]
Primary stimuli	IFN‐γ, LPS	IL‐4, IL‐13	[41, 64]
Biomarkers	iNOS, IL‐12	Arg1, Ym1	[65, 66]
Surface markers	CD86, MHC II	CD206, CD163	[39, 63, 67]
Secretions	Pro‐inflammatory cytokines (IL‐12, TNF‐α)	Anti‐inflammatory cytokines (IL‐10, TGF‐β)	[68, 69, 70]
Functions	Microbicidal activity, tumor suppression	Tissue repair, resolution of inflammation	[40, 68, 71]
Metabolic pathway	Glycolysis	Oxidative phosphorylation, fatty acid oxidation	[72, 73]
Enzymatic proteins	iNOS, NADPH oxidase	Arg1, Fizz1	[74, 75, 76]

Abbreviations: Arg1, arginase 1; CD86, cluster of differentiation 86; CD163, cluster of differentiation 163; CD206, cluster of differentiation 206; Fizz1, found in inflammatory zone 1; IFN‐γ, interferon gamma; IL‐4, interleukin 4; IL‐10, interleukin 10; IL‐13, interleukin 13; iNOS, inducible nitric oxide synthase; IL‐12, Interleukin 12, LPS, lipopolysaccharide; MHC II, major histocompatibility complex class II; NADPH oxidase, nicotinamide adenine dinucleotide phosphate oxidase; TNF‐α, tumor necrosis factor alpha; TGF‐β, transforming growth factor beta, Ym1: chitinase‐like protein 1 (CLP1).

## 7 Macrophage Functions and Polarization in Psoriasis: Emphasizing the Role of M2 Phenotype

Macrophages play a crucial role in the development and chronic nature of psoriasis, primarily through their varied functional states, recognized as polarization [93, 94]. In psoriatic lesions, macrophages exhibit the capacity to undergo polarization into either M1 or M2 phenotypes, each contributing uniquely to the progression of the disease. Biomarkers such as CD163, expressed on M2 macrophages, are valuable in assessing disease activity and therapeutic responses in psoriasis [95]. M1 macrophages have distinct properties that are determined by their tissue environment. Inflammatory cytokines including TNF‐α, IL‐15, IL‐23, and IL‐1β play a crucial role in the immune response to infections [40]. They also have a greater ability to deliver antigens, which is essential for rousing other immune cells. Furthermore, M1 macrophages produce larger quantities of reactive nitrogen and oxygen intermediates (RNI and ROI), which help to destroy infections. Finally, they produce high levels of IL‐12, which aids in the Th1 polarization of CD4+ cells. Overall, M1 macrophages are potent effector cells that produce copious amounts of pro‐inflammatory cytokines while also exhibiting microbicidal and cytotoxic properties [40, 96]. Toll‐like receptors (TLR7 and TLR8) on macrophages are activated by keratinocyte (KC) ferroptosis, leading to the production of antimicrobial peptides (AMPs) like DNA and LL‐37‐bound self‐RNA. This activation helps macrophages transition to the M1 phenotype [97, 98]. High Mobility Group Box 1 (HMGB1), released from necrotic cells, acts as a warning signal that enhances M1 polarization. This excessive M1 polarization in psoriatic lesions amplifies pro‐inflammatory cytokine release, contributing to the chronic nature of psoriasis and worsening disease severity [94, 97]. Extracellular vehicles (EVs) play a role in facilitating macrophage infiltration and M1 polarization by delivering danger signals and chemokines like CX3CL1, which interacts with macrophage CX3CR1 receptors [97, 99]

Conversely, M2 macrophages play a role in tissue repair and anti‐inflammatory responses [71, 72]. They are polarized by Th2 cytokines like IL‐4 and IL‐13, exhibiting anti‐inflammatory and immuneregulatory properties [35, 68]. Activation of transcription factors like STAT3, STAT6, IRF‐4, and PPAR‐γ enable M2 macrophages to produce anti‐inflammatory cytokines such as IL‐10 and TGF‐β [68, 100]. In autoimmune disorders, reduced phagocytic activity of macrophages hinders the clearance of apoptotic cells, leading to increased inflammation by promoting the generation of autoimmune antigens and antibodies [80, 101]. Macrophages also contribute to the migration and abnormal activation of T cells, favoring Th1/Th17 differentiation over Treg differentiation. This dysregulation ultimately triggers B cell activation Imbalances between M1 and M2 macrophages are associated with autoimmune diseases, with skewed M1 activation resulting in the release of pro‐inflammatory cytokines like IL‐6, iNOS, TNF‐α, and IL‐1β, exacerbating organ inflammation [80, 102]. Reduced M2 polarization leads to a lack of anti‐inflammatory cytokine production and compromised immunological tolerance [102, 103]. In conditions such as Psoriasis, disrupted M2 macrophage polarization not only affects vascular proliferation and fibrosis but also intensifies the disease's severity [80, 104].

## 8 Recent Research on the Role of M2 Macrophages in Psoriasis

In psoriasis, M2 macrophages are crucial for reducing inflammation and aiding in skin lesion healing. Dysregulation of macrophage polarization not only exacerbates local inflammation but may also contribute to systemic effects and comorbidities such as cardiovascular diseases and depression and psoriatic arthritis, as evidenced by the psoriatic march and the role of osteoclasts in joint damage. The relationship between obesity and psoriasis onset, as well as the role of comorbidities in exacerbating psoriasis severity, should also be considered [95, 105]. Monitoring the rise of M2 macrophages or the decline of M1 macrophages can help guide treatment strategies for psoriasis, a condition linked to overactive TLRs, specifically TLR7, TLR8, and TLR9 [95]. The study aimed to understand immune mechanisms in psoriasis, revealing that M2 macrophages dominate the dermis of psoriatic skin. These macrophages (RM 3/1 + 3, MS‐1 + /−, 25F9−) help reduce inflammation and regulate immunity. Psoriasis shows a decrease in classical macrophage phenotype due to T‐helper type 1 cytokines [106]. Another study examined CD163(+) cells and soluble CD163 level in Cutaneous T cell Lymphoma (CTCL) and Atopic Dermatitis (AD), and psoriasis‐affected skin. Results indicated increased CD163(+) cells and higher serum sCD163 levels in these conditions, suggesting a link to disease severity and their potential as biomarkers for guiding personalized treatment approaches in psoriasis. M2 macrophages, known for producing IL‐10 and expressing CD163 and mannose receptors, may play a crucial role. Future investigations on CD163(+) cells could unveil new treatment targets and disease mechanisms [107]. Patients with Spondyloarthritis (SpA), including Psoriatic Arthritis (PsA), showed higher levels of M2 markers in their synovial fluid compared to those with Rheumatoid Arthritis (RA). However, dysregulated M2 macrophages in PsA can contribute to tissue fibrosis, emphasizing the need for targeted therapeutic strategies. However, there was no significant increase in M2 polarizing cytokines. Levels of M1‐derived pro‐inflammatory mediators decreased significantly in SpA, particularly in PsA, while M2 mediators remained stable. This suggests a unique local inflammatory environment in SpA compared to RA, with a specific reduction in pro‐inflammatory M1 mediators like TNF‐α and IL‐1beta, which play a role in SpA pathogenesis, including PsA [108]. The study investigated the impact of the PSORI‐CM02 formula on imiquimod‐induced psoriasis and macrophage infiltration and polarization. Results showed that PSORI‐CM02 effectively reduced skin lesions and decreased macrophage infiltration in mice by modulating M1 and M2 macrophage mediators. In vitro experiments confirmed that PSORI‐CM02 inhibited M1 macrophage proliferation while promoting M2 macrophage proliferation. The formula also influenced the production of key cytokines and markers associated with M1 and M2 macrophages through the STAT1 and STAT6 pathways, highlighting its potential in psoriasis treatment. Similarly, biologic therapies targeting IL‐17 and IL‐23 could be explored as tools for modulating macrophage polarization to enhance anti‐inflammatory responses [109]. Researchers discovered that coculturing bone marrow–derived macrophages with Treg‐of‐B cells led to an increase in M2‐associated chemicals. Treg‐of‐B cells activate STAT6 to promote the polarization of M2 macrophages, resulting in reduced production of TNF‐α and IL‐6 in the inflammatory environment. Treatment with Treg‐of‐B cell‐induced M2 macrophages effectively alleviated clinical symptoms of psoriasis in an IMQ‐induced psoriatic mouse model. These findings suggest that Treg‐of‐B cells can activate M2 macrophages through STAT6, offering a potential cell‐based treatment approach for psoriasis [110]. The study aimed to identify diagnostic biomarkers for psoriasis by analyzing genes associated with atherosclerosis. Four genes (SELP, CD93, VAV1, and IL2RG) were identified as the most reliable diagnostic indicators for psoriasis. Additionally, a high concentration of infiltrating immune cells, including M2 macrophages, was observed in psoriatic tissues. These diagnostic markers were closely associated with immune cell infiltration, immune responses, and lipid metabolism in psoriasis [111]. IL‐35 reduces inflammatory cytokine release in psoriasis and alters the M1/M2 macrophage ratio. In various psoriasis models such as HaCaT cells, K14‐VEGF‐A transgenic mice, and imiquimod‐induced psoriasis mice, IL‐35 has been shown to inhibit the production of pro‐inflammatory cytokines such as IL‐6, CXCL8, and S100A7, while increasing IL‐10 and regulatory T cells (CD4 + IL‐10+ and CD4 + CD25+Foxp3 + ). IL‐34 also decreases CD4 + IL‐17 + T cells and shifts the macrophage balance towards an anti‐inflammatory M2 phenotype. Compared to dexamethasone, IL‐35 demonstrates longer therapeutic effectiveness, making it a promising treatment for chronic inflammatory skin diseases [112]. Additionally, the cytokine METRNL is associated with alternatively activated macrophages and barrier tissues, being expressed in skin, activated macrophages, and mucosal tissues. METRNL is produced by M2‐like macrophages and is overexpressed in skin conditions like psoriasis, prurigo nodularis, actinic keratosis, and atopic dermatitis, as well as in rheumatoid arthritis synovial membranes, suggesting its role in influencing innate and acquired immune responses [113].

In psoriatic lesions, M2 macrophages express fewer pro‐inflammatory cytokines compared to M1 macrophages. Monitoring M1/M2 ratios and their role in regulating TLRs 7‐9 could help understand inflammatory responses in psoriasis, particularly in chronic immune dysfunction. Treatment with a TLR 7 agonist led to an increase in the M1/M2 ratio, indicating a shift towards M1 polarization. M1 macrophages have higher levels of pro‐inflammatory cytokines and TLRs 7‐9 due to activation by internal and external ligands of TLRs 7‐9. This suggests that M2 macrophages may regulate the inflammatory response triggered by endosomal TLRs in psoriatic inflammation, a key contributor to the chronic immune dysfunction observed in psoriasis [114]. Acitretin, a psoriasis treatment targeting keratinocytes, reduced the frequency of myeloid‐derived suppressor cells (MDSCs) in psoriasis patients and mice. Acitretin promoted the differentiation of MDSCs into CD206 + M2 macrophages and dendritic cells by increasing glutathione synthase (GSS) expression and glutathione (GSH) accumulation in MDSCs. This effect was mediated by acitretin activating extracellular signal‐regulated kinase 1/2 to regulate GSS expression. These findings suggest a novel mechanism by which acitretin improves psoriasis by promoting MDSC differentiation into immune cells [115]. Tofacitinib inhibits the differentiation of monocytes into regulatory M2 macrophages and promotes the development of inflammatory M1‐like macrophages. It also influences the differentiation of human dendritic cells, leading to a shift towards M1‐like phenotypes with reduced M2 markers. These findings suggest that tofacitinib has a pro‐inflammatory effect on immune cell differentiation [116]. In obese psoriasis patients, pioglitazone therapy did not significantly affect CD163+ (M2‐like) macrophage infiltration, indicating that its therapeutic impact is specifically targeted at CD68+ (M1‐like) pro‐inflammatory macrophages. This selective modulation demonstrates that pioglitazone can reduce inflammation in psoriasis by altering the balance of M1 and M2 macrophage phenotypes towards a more anti‐inflammatory state, without directly affecting M2 macrophage levels in the affected skin [117].

## 9 Discussion

Psoriasis, characterized by red, scaly patches on the skin, significantly affects quality of life. Genetic factors such as HLA‐Cw6 and CARD14 mutations, along with environmental factors, contribute to the complex development of psoriasis [15, 21, 24]. Early diagnosis and personalized treatment are essential for managing symptoms, halting disease progression, and enhancing overall well‐being. This is particularly important as psoriasis is associated with systemic comorbidities such as cardiovascular disease, diabetes, and depression, which significantly impact patient health [18, 25, 26, 27]. In psoriasis, macrophages have dual roles: M1 macrophages promote inflammation, while M2 macrophages facilitate tissue repair and anti‐inflammatory responses. Additionally, M2 macrophages shape T cell differentiation (e.g., Th1, Th2, Th17) and interact with neutrophils, which strongly infiltrate psoriatic lesions, emphasizing their critical role in immune regulation [68, 69]. Imbalance in M1/M2 polarization can exacerbate psoriasis and hinder immune tolerance, highlighting the critical role of macrophage phenotypes in disease management [69, 80]. In this review, we focused on the significant role of M2 macrophages in psoriasis. With the increasing prevalence of psoriasis and its global health impact, understanding the complex relationship between immune responses, macrophage polarization, and disease progression is crucial. Our investigation aimed to shed light on how M2 macrophages influence the pathophysiology of psoriasis, with a particular emphasis on their potential as a therapeutic target. Recent research highlights underscored the critical role of M2 macrophages in managing psoriasis by reducing inflammation and promoting tissue repair. Several studies have elucidated the functions of M2 macrophages in psoriatic conditions and their therapeutic potential. Recent research emphasizes the crucial role of M2 macrophages in psoriasis. These macrophages are predominant in the psoriatic dermis, helping in inflammation reduction and immune regulation [106]. Studies have linked CD163(+) M2 macrophages to the progression of cutaneous T cell lymphoma [107]. In psoriatic arthritis, an increase in M2 markers and a decrease in pro‐inflammatory M1 mediators indicate a distinct inflammatory profile. However, dysregulation of M2 macrophages can contribute to pathological fibrosis, worsening joint damage and chronic inflammation, which underscores the need for targeted therapies to balance macrophage phenotypes [108]. Therapeutic interventions like PSORI‐CM02 and biologic therapies targeting pathways such as IL‐17 and IL‐23 can enhance M2 macrophage function, offering promising approaches to alleviate psoriatic symptoms [109]. Treatments involving Treg‐of‐B cells activate M2 macrophages via the STAT6 pathway, reducing TNF‐α and IL‐6 production and alleviating psoriasis symptoms in mice. This interaction between Treg‐of‐B cells and M2 macrophages illustrates the potential for cell‐based approaches to modulate immune responses in psoriatic lesions by regulating cytokine secretion and immune cell differentiation [110]. Early diagnosis and personalized treatment are essential for managing symptoms, halting disease progression, and enhancing overall well‐being. This is particularly important as psoriasis is associated with systemic comorbidities such as cardiovascular disease, diabetes, and depression, which significantly impact patient health [111]. IL‐35 shifts the M1/M2 balance towards anti‐inflammatory M2 in psoriasis [112]. The expression of METRNL highlights the involvement of M2 macrophages in immune modulation across various skin conditions [113]. While tofacitinib promotes inflammatory M1‐like macrophages, while pioglitazone selectively reduces of M1 macrophages [116, 117]. Research suggests that an increase in M2 macrophage infiltration or a decrease in M1 macrophage infiltration could offer insights into potential psoriasis treatment approaches related to overactive Toll‐like receptors (TLRs). These findings underline the importance of innate immune mechanisms in driving the chronic inflammatory responses in psoriasis [114]. Acitretin promotes the growth of MDSCs into CD206 + M2 macrophages and dendritic cells, contributing to immune cell healing [115]. Enhancing M2 macrophage function is a promising strategy for treating psoriasis, necessitating further targeted research efforts (Table 2).

**Table 2:** Recent research on the role of M2 macrophages in psoriasis.

Study design	Sample Size	Macrophage Phenotype	Role of M2 Macrophages	Intervention/Treatment	Outcome	Ref
Descriptive‐analytical	psoriatic skin lesions (*n* = 21), allergic contact dermatitis (*n* = 4) normal skin (*n* = 2)	RM 3/1 + + + , MS‐1 + /‐, 25F9‐ (Type II macrophages)	Reducing inflammation and regulating immunity reducing the classical macrophage phenotype due to T‐helper type 1 cytokines	The use of a panel of monoclonal antibodies (mAb)	Immunohistochemical identification of type II alternatively activated dendritic macrophages (RM 3/1 + 3, MS‐1 + /‐, 25F9‐) in psoriatic dermis	[106]
Clinical studies	Lesional skin and serum samples from patients with CTCL, AD, or psoriasis, and normal controls	CD163(+) and CD68(+)	Produce IL‐10, express CD163, associated with poor prognosis in CTCL	Topical steroid and ultraviolet light (in CTCL)	CD163(+) cell numbers and serum sCD163 levels associated with disease progression in CTCL, AD, and psoriasis	[107]
Comparative study	47 patients with non‐psoriatic peripheral spondylarthritis (SpA), 55 with Rheumatoid arthritis (RA), 15 with psoriatic arthritis (PsA)	CD163+ macrophages (M2 phenotype)	Preferential expression of M2 markers (CD163, CD200R) in SpA synovial fluid compared to RA; assessment of local inflammatory milieu	Testing synovial fluid (SF) from SpA and RA on macrophage polarization in vitro	Different SF cytokine profiles in SpA and RA; reduced M1‐derived mediators in SpA synovitis compared to RA	[108]
Experimental study	Male BALB/C mice weighing 18–22 g and SD rats weight 220–250 g – Control group – Model group – Methotrexate group – PSORI‐CM02 high dose group – PSORI‐CM02 middle dose group – PSORI‐CM02 low dose group	Decreased M1 mediators, increased M2 mediators	Elevated M2 macrophage proliferation contributes to inflammation resolution and tissue repair in the context of psoriasis.	PSORI‐CM02 formula	Improved skin lesions, reduces macrophage infiltration, modulated cytokine expression related to M1 and M2 macrophages, regulated STAT1 and STAT6 pathways	[109]
Coculture experiment	Six‐to‐eight weeks old male BALB/c mice	M2‐like macrophages	Polarization induced by Treg‐of‐B cells (Foxp3‐)	Coculture with Treg‐of‐B cells under LPS/IFN‐γ stimulation	M2‐like macrophages polarized by Foxp3‐ Treg‐of‐B cells ameliorate imiquimod‐induced psoriasis.	[110]
Bioinformatics analysis and experimental	GSE78097: 27 psoriasis, 6 normal. GSE28829: 16 advanced atherosclerosis, 13 early atherosclerosis. GSE14905: 33 psoriasis, 21 normal. GSE57691: 9 atherosclerosis, 10 normal	M0, M2, B‐cell memory	Involved in immune response in psoriasis	Analysis of microarray datasets, immune cell infiltration, and lincRNA‐miRNA‐mRNA network construction	Identification of SELP, CD93, IL2RG, and VAV1 as potential biomarkers for psoriasis	[111]
Coculture experiment	human keratinocyte cell line (HaCaT) mouse model, keratin 14 (K14) mouse model ‐ vascular endothelial growth factor A (VEGF‐A)‐transgenic (Tg) mouse model, and an imiquimod‐induced psoriasis mouse model.	M2 macrophages	Decreases M1/M2 macrophage ratio	IL‐35 plasmid coated with cationic liposomes	IL‐35 Decelerates the Inflammatory Process by Regulating Inflammatory Cytokine Secretion and M1/M2 Macrophage Ratio in Psoriasis	[112]
Observational and Experimental	Human samples (various skin conditions), Mouse models (BALB/C and C57BL/6)	M2‐like macrophages, AAMs (Alternatively Activated Macrophages)	METRNL is expressed by AAMs and M2‐like macrophages, involved in immune responses.	Treatment of macrophages with IL‐4, IL‐13, LPS, PGE2; Analysis of gene expression in human and mouse tissues	METRNL expression associated with barrier tissues, skin diseases (psoriasis, prurigo nodularis, actinic keratosis, atopic dermatitis), and alternatively activated macrophages	[113]
Observational and Experimental	Human samples: (THP‐1 cells, a line of human monocytic cells derived from an acute monocytic leukemia patient) Animal samples: Balb/c mice	M1 and M2	M2 macrophages produce anti‐inflammatory cytokines	Imiquimod (TLR 7 agonist), IFN‐γ, IL‐4	Increased M1 macrophage polarization and cytokine expression Amelioration of psoriatic response upon macrophage depletion	[114]
Clinical study and	77 patients with plaque psoriasis 30 healthy controls 20 psoriasis patients’ skin 9 healthy controls' skin	Expansion of MDSCs and M‐MDSCs in psoriasis patients	Reduction in MDSCs and M‐MDSCs Promotion of M2 macrophages and dendritic cells	Acitretin (30 mg/d for 8 weeks in patients, 5 mg/kg daily in mice)	Improvement in PASI scores Decrease in MDSCs and M‐MDSCs Enhanced differentiation into M2 macrophages and dendritic cells Upregulation of GSS and GSH in MDSCs	[115]
Experimental study	BALB/c female mice, 8‐week‐old	Evaluation of skin tissues	Effects on MDSCs and M‐MDSCs	Acitretin (5 mg/kg daily, oral administration)	Histological assessment of skin tissues Measurement of MDSCs and M‐MDSCs in mouse models	
Experimental study	17 healthy donors	Reduced differentiation into immature DCs	Favors M1‐like macrophage phenotype	Tofacitinib (10 µM)	Inhibited differentiation of monocytes into M2 macrophages, Enhanced M1 macrophage development, Altered response to maturation stimuli (LPS and IFNγ) as assessed by qPCR.	[116]
Clinical study	6 obese psoriatic patients	CD68+ (M1‐like) CD163+ (M2‐like)	Reduction in CD68+ macrophages	Pioglitazone (15, 30, and 45 mg daily for 6 months)	Decreased CD68+ macrophage infiltration in psoriasis‐affected skin, No significant change in CD163+ macrophages, Improvement in skin morphology with higher pioglitazone doses.	[117]

Abbreviations: AAMs, alternatively activated macrophages; AD, atopic dermatitis; CD68, cluster of differentiation 68; CD93, cluster of differentiation 93; CD163, cluster of differentiation163; CTCL, cutaneous T‐cell lymphoma; Foxp3, forkhead box P3; GSS, glutathione synthetase; GSH, glutathione; HaCaT, human keratinocyte cell line; IFN‐γ, interferon gamma; IL2RG, interleukin 2 receptor gamma; IL‐35, interleukin 35; mAb, monoclonal antibody; MDSCs, myeloid‐derived suppressor cells; METRNL: meteorin‐like protein; M‐MDSCs, monocytic myeloid‐derived suppressor cells; LPS, lipopolysaccharide; SpA, spondyloarthritis; K14, keratin 14; PASI, psoriasis area and severity index; PGE2, prostaglandin E2; PsA, psoriatic arthritis; qPCR, quantitative polymerase chain reaction; RA, rheumatoid arthritis; SELP, selectin P; SF, synovial fluid; STAT1, signal transducer and activator of transcription 1; STAT6, signal transducer and activator of transcription 6; TLR 7, toll‐like receptor 7; Treg‐of‐B cells, regulatory T cells derived from B cells; VAV1, vav guanine nucleotide exchange factor 1; VEGF‐A, vascular endothelial growth factor A.

## 10 Conclusions

The study emphasizes the significant role of M2 macrophages in psoriasis development and treatment. Balancing M1 and M2 macrophages through medical or natural interventions, including biologic therapies targeting IL‐17 and IL‐23, shows promise for managing psoriasis and other skin inflammations. Strategies that enhance M2 polarization while suppressing M1 inflammatory responses could enhance patient outcomes and address the systemic impacts of psoriasis, including its comorbidities such as cardiovascular disease and depression by addressing the systemic impacts of psoriasis. Advancing our knowledge of macrophage biology in psoriasis, particularly the role of biomarkers like CD163, may unveil new therapeutic targets and pathways for personalized treatment and improved patient care, potentially leading to more effective treatments for immune cell dysregulation by addressing the innate immune mechanisms and their role in chronic inflammation, thereby improving the quality of life for those with this chronic skin condition.

## Author Contributions

**Ahmed Hussein Hasan Alshihmani:** methodology, project administration, writing – original draft. **Mahmoud Mahmoudi:** supervision. **Ramiar Kamal Kheder:** writing – review and editing. **Afsane Fadaee:** project administration, writing – review and editing. **Seyed‐Alireza Esmaeili:** conceptualization, supervision.

## Ethics Statement

The current study is review article and manuscript complies with the Ethical Rules applicable for this journal.

## Conflicts of Interest

The authors declare no conflicts of interest.

## Data Availability

The data set(s) supporting the conclusions of this article is (are) included within the article (and its additional file(s)).
